# Isotactic-Polypropylene/Atactic-Polystyrene Miktoarm Star Copolymers: Synthesis and Aggregation Morphology

**DOI:** 10.3390/polym11101574

**Published:** 2019-09-27

**Authors:** Yuanjie Wang, Xinzhi Liu, Liying Liu, Hui Niu

**Affiliations:** State Key Laboratory of Fine Chemicals, Department of Polymer Science and Engineering, School of Chemical Engineering, Dalian University of Technology, Dalian 116024, China; yuanjiewang@iccas.ac.cn (Y.W.); wolong@mail.dlut.edu.cn (X.L.); lxhn@mail.dlut.edu.cn (L.L.)

**Keywords:** miktoarm star copolymer, isotactic-polypropylene, atactic-polystyrene, aggregation morphology

## Abstract

In this work, a series of isotactic-polypropylene/atactic-polystyrene (*i*PP/*a*PS) miktoarm star copolymers, P_x_S_y_, was synthesized via an arm-first approach. Varied star macromolecule architectures were fabricated by designing the arm length and the arm numbers (x and y). These miktoarm stars were able to form micelles in selective solvent (*N*,*N′*-dimethylformamide (DMF)), in which the insoluble *i*PP arms formed the core and the soluble *a*PS arms formed the shell. The miktoarm polymers aggregated to micro-nanoscale binary structures (MNBSes) in the casting process, and their morphologies, including the MNBS shape and size, were greatly influenced by the P_x_S_y_ architectures. The MNBSes endowed the material surface with superhydrophobic performance with a water contact angle of 157.0° and a sliding angle of 1.5°.

## 1. Introduction

The design of polymer topology provides convenient ways to control the properties of materials. In the past decades, the self-assembly of linear block copolymers in selective solutions has been a very important area in both experimental and theoretical research [1,2,3,4,5,6]. This interest is mainly due to the application of block copolymer self-assembly in functional materials and nanoscale devices. For instance, amphiphilic diblock copolymers performing various self-assembly behaviors in selective solvents have offered opportunities for novel bionic applications, imitating the vast extraordinary morphologies on biological surfaces in nature that exhibit peculiar performances such as super-water-repellent properties [7,8,9]. Xu et al. [10] easily fabricated a superhydrophobic surface with a bionic micro-nanoscale binary structure (MNBS) from a common isotactic-polypropylene/poly(methyl methacrylate) diblock copolymer (*i*PP-*b*-PMMA) based upon the principle of micelles, where diblock copolymers usually form in selective solvent. In *N*,*N′*-dimethylformamide (DMF), the *i*PP-*b*-PMMA formed a micelle solution, and each micelle was composed of an insoluble *i*PP core and a soluble PMMA shell; thus, through a one-step casting process, a material surface with bionic superhydrophobicity was obtained. The surface possessed an MNBS similar to that of a lotus leaf, where every microscale papilla (1–2 μm) is covered by nanoscale papillae (50–200 nm) [8,11,12]. This strategy provided a replacement for traditional complex superhydrophobic surface preparation technology, which combines surface energy reduction [13] with surface roughness improvement [14,15].

Star-like copolymers are branched macromolecules that are connected by several linear arms jointed at one junction point. They have hydrodynamic radii smaller than those of linear copolymers with similar molecular weights [1,5]. They have a compact and three-dimensional structure with a large surface area. Heteroarm star copolymers, including block-arm copolymers ((AB)_n_) and miktoarm copolymers (ABC or A_x_B_y_) exhibit many unique micellar properties different from block copolymers, which has been discussed in a series of experimental studies [16,17,18]. Take miktoarm stars A_x_B_y_, for example: compared to the linear diblock copolymer AB, A_x_B_y_ exhibits more complicated behaviors. Pispas et al. [19,20] compared the polystyrene/polyisoprene (S_x_I_y_) miktoarm stars SI_2_ and S_2_I to diblock SI and found that the aggregation numbers of their micelles in *n*-decane increased in the order SI_2_ < S_2_I < SI, indicating that the influence of star architecture on micellization is more complex than in the corresponding linear block copolymer.

Since Xu’s pioneering work [10] proved that by benefiting from the crystallinity and solvency resistance of *i*PP, even an ordinary diblock copolymer (*i*PP-*b*-PMMA) could display advantages in MNBS surface fabrication, we could expect that *i*PP miktoarm star copolymers would exhibit a more diversified performance due to the complexity of the miktoarm star copolymer architecture. Although a series of well-defined *i*PP block and graft copolymers are emerging from progress in propylene coordination polymerization technology [21,22,23], the synthesis of miktoarm star-shaped *i*PP has not been reported. This is mainly due to an unavoidable chain transfer reaction at the *i*PP propagating end and an inert feature at the *i*PP initiating end, resulting in difficulties in *i*PP “arm” fabrication.

Recently, we reported an efficient route to synthesize *i*PP star-like homopolymers through an arm-first approach via a designed styryl-capping procedure in propylene polymerization. In this approach, a reactive *i*PP precursor containing a methoxysilane terminal end was fabricated for the first time [24]. Based upon this technique, in the present work a series of isotactic-polypropylene/atactic-polystyrene (*i*PP/*a*PS) miktoarm star copolymers P_x_S_y_ (x and y represent the arm numbers) were synthesized for the first time, and the influence of *i*PP/*a*PS miktoarm star copolymer architecture on their aggregation morphology and surface superhydrophobic properties was investigated in detail.

## 2. Materials and Methods

### 2.1. Materials and Measurements

All O_2_ and moisture-sensitive manipulations were carried out inside an argon-filled vacuum atmosphere dry-box. Chemical pure-grade tetrahydrofuran (THF), hexane, and toluene were refluxed over Na/benzophenone. *N*,*N′*-dimethylformamide (DMF), 4-chlorostyrene and SiCl_4_ were purchased from Aladdin Company (Shanghai, China). The metallocene catalyst *rac*-C_2_H_4_(Ind)_2_ZrCl_2_ was from J&K Chemical (Beijing, China). Methylaluminoxane (MAO) was purchased from Energy Chemical (Shanghai, China) and was dried under vacuum to remove trimethylaluminum (TMA). The resulting dry MAO (dMAO) was diluted in toluene. The free radical initiator 2,2′-azobis(isobutyronitrile) (AIBN) from Energy Chemical was purified through recrystallization twice from methanol. Bromobenzene, CS_2_, and stannous octoate (SnOct_2_) were obtained from Energy Chemical and were used as received; and 4-(chloromethyl)phenyl trimethoxysilane (90%) was purchased from Alfa Aesar (Tianjin, China) and was used as received. Styrene was purified by distillation under reduced pressure to remove the inhibitor. Polymerization-grade propylene was purchased from Dalian Guangming Gas Company (Liaoning, China).

All ambient-temperature ^1^H-NMR spectra were measured in chloroform-*d* at 25 °C with a Bruker Avance III-500 instrument (Karlsruhe, Germany). All high-temperature ^1^H-NMR and ^13^C-NMR spectra were recorded in 1,1,2,2-tetrachloroethane-*d*_2_ at 100 °C with a Vaian DLG 400 instrument (PaloAlto, CA, USA). Polymer characterizations with high-temperature gel permeation chromatography (GPC) were carried out on PL-GPC220 equipment (South Queensferry, UK). A differential refractive index detector, a capillary viscometer, and a two-angle light-scattering (LS) detector were equipped. The detecting angles of the light-scattering detector were 15° and 90°. Here, 1,2,4-trichlorobenzene was used as the eluent, and the test was carried out at 150 °C with a flow rate of 1.0 mL/min. Narrow molecular-weight-distributed polystyrene samples were used for calibration as standards. The melting curves of the polymers were measured by differential scanning calorimetry (DSC, TA Q2000, New Castle, PA, USA) with a heating and cooling rate of 10 °C/min under a nitrogen atmosphere. The second-scan data were collected to remove any heat history. The polymer morphology was examined by scanning electron microscopy (SEM, Hitachi SU8200 instrument, Tokyo, Japan), and the acceleration voltage was 5 kV. All samples were Pt-coated before SEM investigation. TEM images were collected using a transmission electron microscopy Hitachi HT7700 EXALENS (Tokyo, Japan) operating at 100 keV. The specimens were prepared by drop-casting the polymer dispersion onto a carbon-coated copper grid. The water contact angle (WCA) was measured with a contact angle detector (Powereach JC2000 D2W, Shanghai Zhongchen Digital Technology Apparatus Co. Ltd, Shanghai, China). The WCA value was obtained with a constant volume of water (4 μL) by averaging five results at different positions on the same sample. The water scroll angle was measured by inclining the specimen on an inclinable platform.

### 2.2. Synthesis of iPP and aPS Arm Precursors

Propylene polymerization was conducted in a 250-mL round-bottomed flask with 50 mL of toluene. A typical reaction involved the sequential addition of 15 mmol of (*p*-vinylphenyl)trichlorosilane (the synthesis route was shown in a previous work [24]), 8 mmol of dMAO, and 4 μmol of metallocene catalyst (*rac*-C_2_H_4_(Ind)_2_ZrCl_2_) into the reaction flask. The polymerization temperature and propylene gas pressure were set at 30 °C and 1 atm, respectively. After 15 min, the polymerization was terminated by pouring the polymer solution into absolute methanol: then *i*PP with a methoxysilane terminal group was obtained. The resulting polymer was washed with methanol, filtered, and dried under vacuum for 24 h at 50 °C. Finally, methoxysilane-terminated *i*PP was obtained.

**Chart 1:**
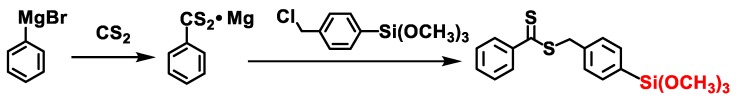
Synthetic route of RAFT agent TBDB.

Atactic polystyrene (*a*PS) was synthesized by reversible addition-fragmentation chain transfer (RAFT) polymerization of styrene in toluene solvent with a monomer volume concentration of 50%. AIBN was used as the initiator and 4-(trimethoxysilyl)benzyl dithiobenzoate (TBDB) (synthesis route is shown in Chart 1 according to the literature [25]) as the RAFT agent. The molar ratio of St/TBDB/AIBN was set as 50/1/0.2, 100/1/0.2, and 150/1/0.2 for the synthesis of PS-1, PS-2, and PS-3, respectively. The reaction was allowed to proceed at 80 °C for 48 h, and the resulting solution was precipitated, washed with methanol, filtered, and dried under vacuum for 24 h at 50 °C. Finally, *a*PS pink powder was obtained.

### 2.3. Synthesis of iPP/aPS Miktoarm Star Copolymers

**Chart 2:**
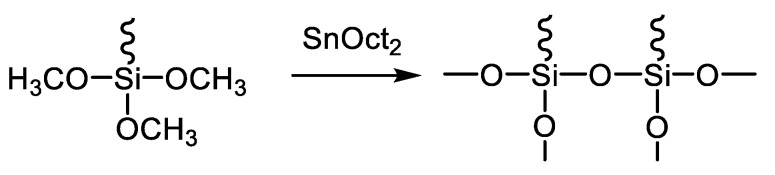
Reaction mechanism of intermolecular elimination catalyzed by SnOct_2_.

Copolymerization was conducted in a 50-mL round-bottomed flask. In a typical miktoarm copolymer (run S-1 in Table 2) synthesis procedure, a mixture of 0.18 g (0.042 mmol) methoxysilane-terminated *a*PS and 0.82 g (0.125 mmol) methoxysilane-terminated *i*PP with 5 wt% stannous octoate (SnOct_2_) [26] was dissolved in 5 mL of toluene under nitrogen. The mixture was stirred for 3 h at 120 °C (the reaction mechanism is shown in Chart 2). The resulting product, the miktoarm star copolymer *i*PP/*a*PS, was precipitated with methanol, then washed with ethanol, and finally dried under vacuum at 50 °C for 24 h: 1.0 g polymer was obtained. The possible *a*PS and star-*a*PS homopolymers were removed through THF-dialyzing at room temperature for 24 h, and the possible *i*PP and star-*i*PP homopolymers were removed by Soxhlet extraction with boiling THF over 10 h (collecting the soluble part to get the miktoarm star copolymer, as shown in Table S1). Finally, the miktoarm star polymer S-1 was obtained.

## 3. Results and Discussion

**Scheme 1:**
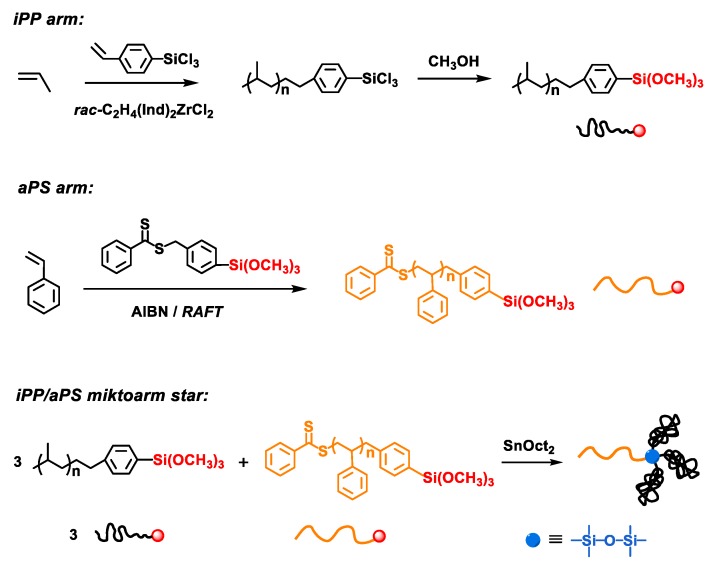
Synthetic route of *i*PP/*a*PS miktoarm star copolymers via arm-first approach.

**Figure 1:**
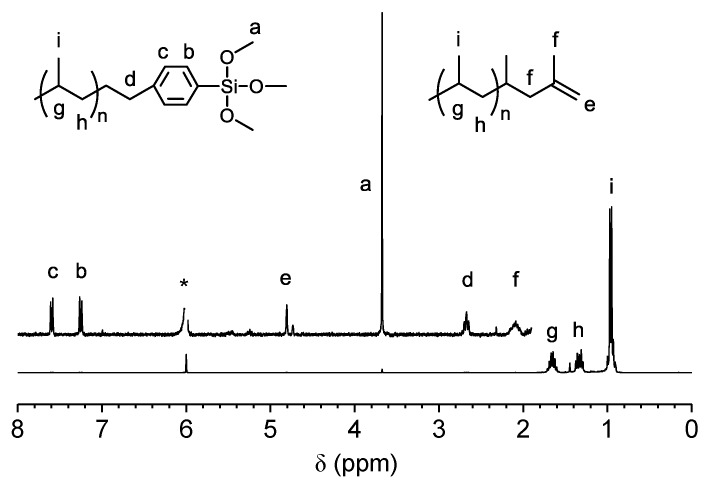
^1^H-NMR spectrum of methoxysilane-terminated *i*PP (*i*PP arm). Solvent (*): 1,1,2,2-tetrachloroethane-*d*_2_.

The methoxysilane-capped *i*PP (*i*PP arm) was synthesized through a facile “two-in-one” approach, as shown in Scheme 1, in which a steric jamming phenomenon (during the styryl 2,1-insertion) was utilized in propylene polymerization, which we successfully implemented in previous work on the synthesis of star-*i*PP [24]. By using TMA-free dMAO, the chain transfer to aluminum was restrained, and thus the terminal groups included two kinds of structures, i.e., vinyl and methoxysilane ends. The ^1^H-NMR spectrum of the methoxysilane-terminated *i*PP obtained is shown in Figure 1. The peak at δ = 4.73–4.80 ppm (peak e) was assigned to a vinyl chain end deriving from β-H elimination, and the signals attributed to the terminal methoxyl and phenyl groups can be observed at *δ* = 3.63 ppm (peak a), *δ* = 7.24 ppm (peak b), and 7.60 ppm (peak c). Then the terminal composition could be analyzed quantitatively. The methoxysilane capping ratio was evaluated quantitatively as 70.4%, and the stereoregularity (presented by the *mmmm* pentad content calculated by ^13^C-NMR [27]) of the *i*PP arm was 82% (Figure S1). The average molecular weight of the *i*PP arm precursor was determined to be 6500 g/mol by GPC.

**Figure 2:**
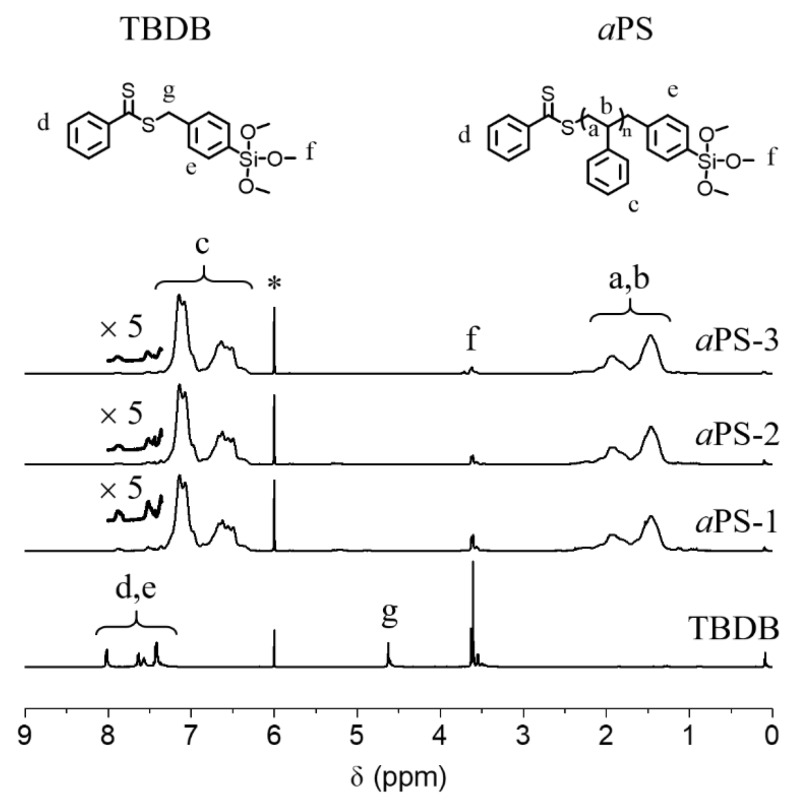
^1^H-NMR spectra of TBDB and methoxysilane-terminated *a*PSes (*a*PS arms). Solvent (*): 1,1,2,2-tetrachloroethane-*d*_2_.

The methoxysilane-terminated *a*PS (*a*PS arm) was synthesized trough a RAFT reaction. AIBN was used as the initiator and TBDB as the RAFT agent, as depicted in Scheme 1. In the TBDB, the C_6_H_5_−(C=S)−S− and −C_6_H_4_− moiety exhibited characteristic ^1^H-NMR resonances (in Figure 2) at *δ* = 8.01 ‒ 7.40 ppm originating from the protons d and e. Peaks at 4.62 ppm (g) and 3.61 ppm (f) represent the protons of the methylene adjacent to the S atom and methoxy groups adjacent to the Si atom, respectively. For the polymers, the ^1^H-NMR spectra presented signals ascribed to proton c at around 7.10 and 6.59 ppm and to protons a and b around 1.2–2.0 ppm, which belonged to the styrene units. Moreover, due to the living feature of RAFT polymerization, three Si(OCH_3_)_3_-terminated *a*PSes with varied number average molecular weights of 4400 (*a*PS-1), 8300 (*a*PS-2), and 11700 (*a*PS-3) were synthesized easily, as displayed in Table 1.

**Figure 3:**
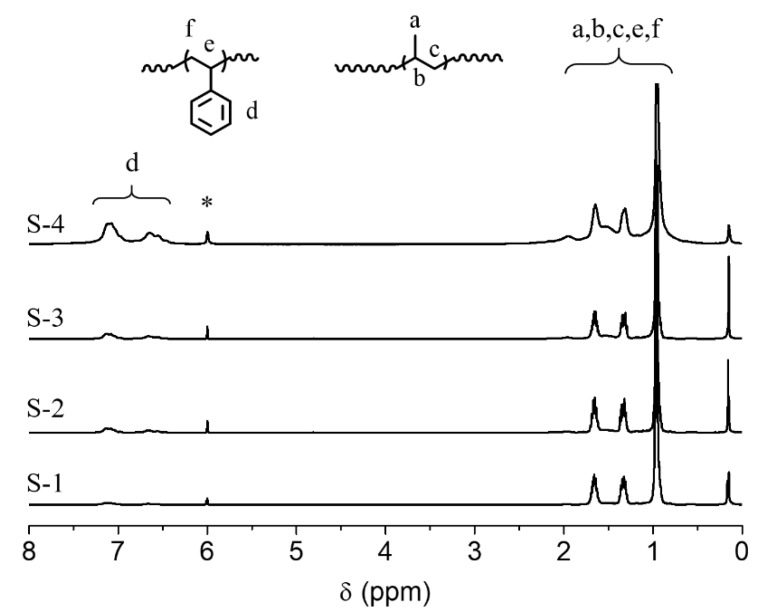
^1^H-NMR spectra of *i*PP/*a*PS miktoarm star copolymers. Solvent (*): 1,1,2,2-tetrachloroethane-*d*_2_.

Having fabricated the *i*PP and *a*PS arms, an intermolecular elimination reaction was carried out to synthesize the miktoarm star copolymers in toluene solution at 120 °C catalyzed with SnOct_2_. A series of miktoarm star copolymers was synthesized by changing the *a*PS arm length and adjusting the feeding ratio of the *a*PS arm and the *i*PP arm, as shown in Table 2. Figure 3 exhibits ^1^H-NMR spectra of the resulting miktoarm star copolymers. The disappearance of *i*PP’s terminal methoxyl group at δ = 3.63 ppm (peak a in Figure 1) and the disappearance of *a*PP’s terminal methoxyl group at 3.61 ppm (peak f in Figure 2) indicated the elimination reaction. Since the possible byproducts (star-*a*PS and star-*i*PP homopolymers) and the unreacted arms were removed by ambient temperature THF-dialysis (to remove the *a*PS) and by boiling THF-Soxhlet extraction (to remove the *i*PP), the composition of the miktoarm star copolymers could be calculated from the ^1^H-NMR results.

**Figure 4:**
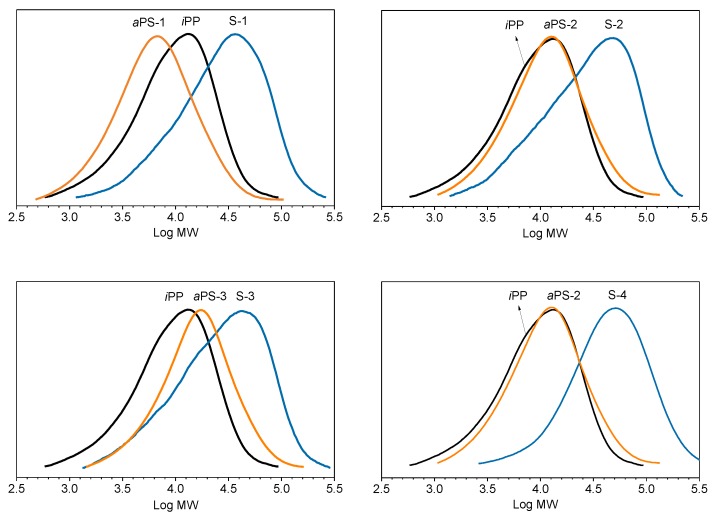
GPC-LS curves of *i*PP/*a*PS miktoarm star copolymers (eluent: 1,2,4-trichlorobenzene; temperature: 150 °C).

A detailed structure in the miktoarm star copolymers was detected by GPC equipped with light-scattering detector GPC-LS (Figure 4). The polymer absolute molecular weights are summarized in Table 2. Combined with the ^1^H-NMR results, the average arm number of *a*PS and *i*PP in each miktoarm star copolymer, *AN_aPS_* and *AN_iPP_*, respectively, was calculated according to the following formulae (Equations (1) and (2)):

(1) $$M_{aPS}\times AN_{aPS}+M_{iPP}\times AN_{iPP}=M_{peak-star},$$

(2) $$\frac{\left(M_{iPP}\times AN_{iPP}\right)/42}{\left(M_{iPP}\times AN_{iPP}\right)/42+\left(M_{aPS}\times AN_{aPS}\right)/104}\times 100=iPP\;\left(mol\%\right),$$

in which *M_a_*_PS_ and *M_i_*_PP_ are the peak molecular weights (*M*_peak_ in Table 1 and Table 2) of *a*PS and *i*PP, respectively; *M*_peak-star_ is the peak molecular weight of the miktoarm star polymer determined by GPC; and *i*PP (mol%) is the propylene content (data shown in Table 2) in the miktoarm star copolymers determined by ^1^H-NMR. In order to give relatively proximate and unified results, the average molecular weight was calculated from the peak molecular weight (*M*_peak_). The total arm number *AN_total_* was the sum of *AN_aPS_* and *AN_iPP_*. The schematics of all of the miktoarm star copolymers are summarized in Table 3.

**Figure 5:**
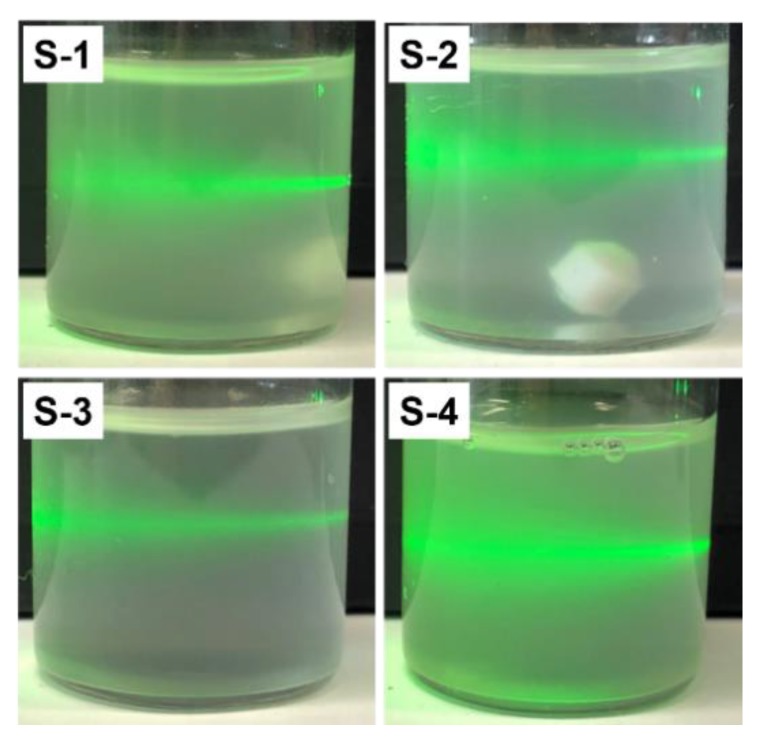
Photographs of *i*PP/*a*PS miktoarm star polymer solutions irradiated by a laser beam after three days.

The weak interaction between selective solvents and insoluble blocks in polymers is the cause of micelle formation. These insoluble blocks are far away from the solvent and aggregate with each other to form micelles [28,29,30]. In this work, DMF was chosen as the selective solvent. The direct dissolution of *i*PP/*a*PS miktoarm star copolymers in DMF was not easy and can be attributed to the crystallinity of *i*PP and the high glass transition temperature (*T*_g_) of *a*PS. The star polymer was first dispersed in DMF, and the mixture was heated to 60 °C to accelerate dissolution. The stock solution was further dispersed by ultrasonic treatment in ambient atmosphere for an additional 2-h period to ensure complete dispersion of the sample. Using the above sample preparation protocol, semitransparent micelle solution was obtained. No polymer precipitation was observed in the miktoarm star solutions after standing for 3 days. A Tyndall effect was observed in the miktoarm star copolymer solutions (Figure 5 and Figure S3).

**Figure 6:**
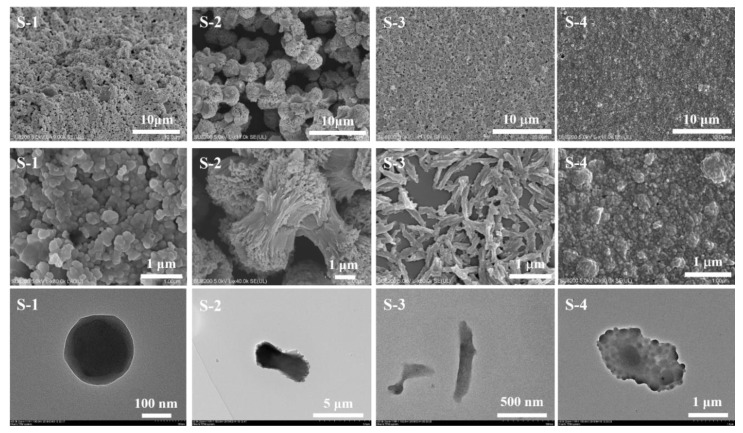
The first and second rows: SEM images of surfaces cast from the *i*PP/*a*PS miktoarm star copolymer solutions; the bottom row: TEM images of the *i*PP/*a*PS miktoarm star copolymers drop-cast from the DMF solution.

The MNBS surfaces were able to be formed through casting of the polymer micelles at relatively low concentrations (*C* = 0.5 mg/mL in selective solvent DMF, much lower than in the *i*PP-*b*-PMMA system, which was reported as 50 mg/mL [10]). Using a dropper to absorb the dispersion, they were dropped on the slide and dried under vacuum at room temperature for 12 h. As the images show in Figure 6, the interplay between the soluble and insoluble parts in these systems, which influence the aggregation morphology, seemed to be rather complex. Polymers S-1 and S-4 presented granular particles with diameters of 100–200 nm, and polymers S-2 and S-3 exhibited a rod-like shape, in which S-2 aggregated to dumbbell-like rods with a diameter of ~3 μm and a length of ~6 μm, whereas S-3 formed tiny rods about 100 nm in diameter and 1 μm in length.

**Figure 7:**
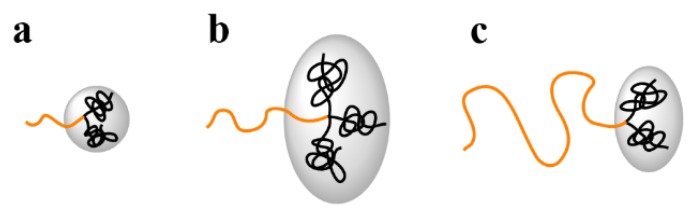
Schematics of *i*PP/*a*PS miktoarm star copolymers with different lengths of soluble *a*PS arms in selective solvent DMF: (**a**) S-1, (**b**) S-2, and (**c**) S-3.

When we compared three miktoarm star polymers with single but different lengths of soluble arms, that is, S-1, S-2, and S-3, an aggregation size order of S-1 (S_short_) < S-3 (S_long_) < S-2 (S_medium_) was observed. This trend was contrary to the common rule of block copolymers. In block copolymers, the aggregation number depends mainly on the insoluble block length rather than the soluble block length [28]. In P*_x_*S*_y_* miktoarm stars, however, as equal numbers (*AN_aPS_* = 1) of the corona segment were connected to two or three *i*PP chains at the same junction point, as depicted in Figure 7a, a short *a*PS arm (S-1) resulted in not only a considerable reduction of the corona thickness and overall size of the micelles, but also the isolation of the particles from each other without apparent entanglement. The formation of an *i*PP core in DMF resulted in the curvature of the core–corona interface toward the two *i*PP arms of the miktoarm copolymer. This is not a comfortable situation for the *i*PP arms, as they have to be accommodated in a small space. Increasing the length of *a*PS improved the solubility of the copolymer and thus favored more elongated structures, as shown in Figure 7b,c. A more elongated/bigger core released some of the crowding [20]. In this way, a larger size of the whole micelle was produced in S-2. However, further extension of the *a*PS length (or volume) contrarily compressed the *i*PP core space and reduced the micelle size (S-3); thus, medium-sized aggregation formed. The coronal chain stretching and copolymer topology around the junction point are important factors that determine micellar size, as well as the aggregation morphology.

Comparing S-2 (P_3_S_1_) and S-4 (P_2_S_2_), which possessed approximately equal overall molecular weights and arm numbers, their aggregation sizes (diameters) increased in the order S-4 (P_2_S_2_) < S-2 (P_3_S_1_). This order was similar to that observed in polystyrene/polyisoprene miktoarm stars (S_x_I_y_) in Pispas’s work [20]. That work compared SI_2_ and S_2_I to diblock SI and found that all samples formed spherical micelles in selective solvent, *n*-decane (for polyisoprene). Aggregation numbers increased in the order SI_2_ < S_2_I < SI. Borisov et al. [17] examined how the A_x_B_y_ miktoarm polymer parameters affect the equilibrium morphology of self-assembling aggregates and the stability range of classical morphologies (sphere, cylinder, and lamella). According to their prediction, an increase in the number of soluble arms in the miktoarm star (keeping the total number of monomer units in soluble blocks constant) would stabilize the spherical geometry of self-assembled micelles, which is coincident with sample S-4 presenting a smaller and more compact spherical morphology than sample S-2 did.

Overall, the most probable cause for the observed surface morphology was a complex combination of copolymer composition, macromolecular architecture, and the solvent. The self-assembly of miktoarm star copolymers in selective solvent is often dominated by competition between the chain-stretching energy in the core, the interfacial energy, and the repulsion energy among the coronal chains [6,31]. Moreover, *i*PP/*a*PS miktoarm star copolymers, to some extent, belong to rod–coil copolymers [16], which contain crystallizable rod-like *i*PP and flexible coil-like *a*PS moieties. Their assembly behaviors might be different from coil–coil S_x_I_y_ miktoarm stars in morphology because of the geometric disparity between the rod and coil segments and the anisotropic interactions between rod blocks, which form crystalline structures.

**Figure 8:**
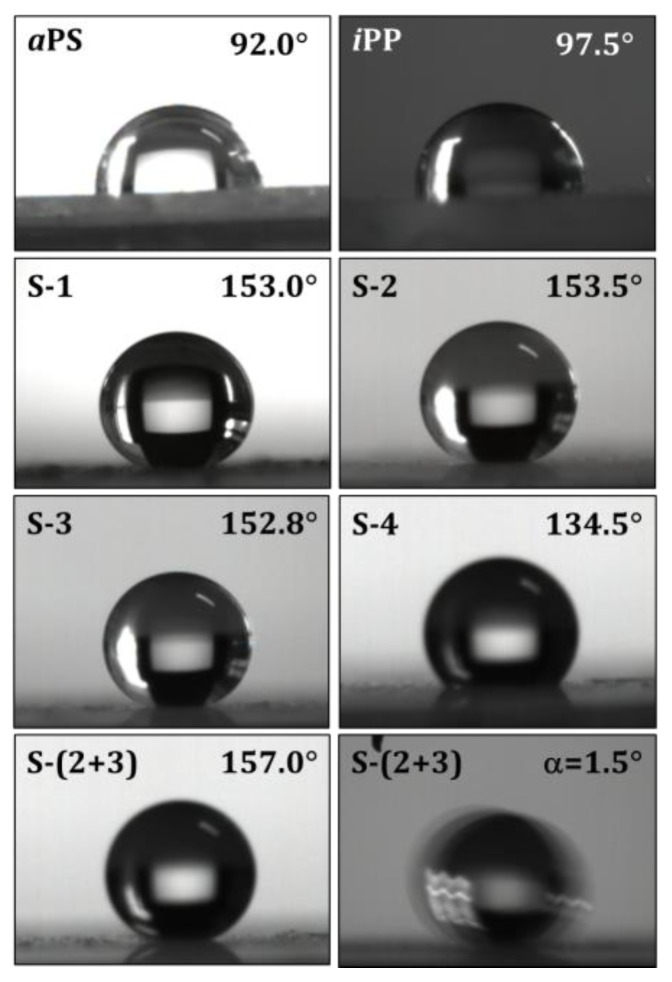
Contact angle of water drops on the surfaces cast from the *i*PP, *a*PS, and *i*PP/*a*PS miktoarm star copolymer solutions and the sliding angle (*α*) of the surface of the S-2/S-3 mixture S-(2 + 3).

The rough surfaces of the *i*PP/*a*PS miktoarm star copolymers presented superhydrophobicity. The contact angles of 4-μL water drops on the surfaces S-1, S-2, S-3, and S-4 were 153.0°, 153.5° 152.8°, and 134.5°, respectively. Although their aggregation sizes varied in orders of magnitude, they all possessed a MNBS morphology of nanoscale papillae covered on each microscale particle: the miktoarm star polymer micellar stacking formed microscale roughness, while the curvature of micelles on the surface of the aggregates provided nanoscale roughness, which endowed these surfaces with a bionic structure similar to a lotus leaf. The MNBS minimized the contact area and created more air space trapped between the water and the surface, which induced the improvement of surface hydrophobicity [13,14]. With the increase of *a*PS arms, the contact angles on the surfaces decreased to 134.5° (S-4), accompanied by adhesion between the microscale particles. However, a surface of S-2/S-3 mixture (1:1, *w*/*w*) cast from DMF exhibited a multilevel scale structure with a high contact angle of 157.0° and a very low sliding angle (α) of 1.5° (sample S-(2 + 3) in Figure 8 and Figure S4), indicating a superhydrophobic and self-cleaning surface was achieved.

## 4. Conclusions

In summary, in this work, a series of *i*PP/*a*PS miktoarm star copolymers (P_x_S_y_) were synthesized via an arm-first approach, and the polymer architectures were fabricated by designing the arm length and the arm number. The polymer morphologies, including the MNBS shape and size, were greatly influenced by the P_x_S_y_ architecture, indicating that the aggregation mechanism for miktoarm star copolymers is a complex combination of polymer composition, molecular architectural effects, and the solvent. The very preliminary nature of the results based on the limited set of experiments became clear. Multilevel bionic MNBS could be obtained from *i*PP/*a*PS miktoarm star copolymer micelles, which produced superhydrophobic properties even though the surface morphology varied with the macromolecular architecture. The experimental results will help in understanding the principles governing the organization of miktoarm star structures constructed in solution and will provide suggestions for designing polymers for specific applications such as super-water-repellent materials or anisotropic particles.

## Figures and Tables

**Table 1:** Characterization of *a*PS arm precursors.

Samples	*M*_n_^a^ (g/mol)	*M*_peak_^a^ (g/mol)	*Đ*
*a*PS-1	4400	6800	1.9
*a*PS-2	8300	12,500	1.8
*a*PS-3	11,700	20,700	1.7

^a^ The average molecular weight *M*_n_, and the peak molecular weight *M*_peak_ were determined by high-temperature GPC with a light-scattering detector.

**Table 2:** Synthesis and characterization of *i*PP/*a*PS miktoarm star copolymers ^a^.

Runs	*a*PS Precursors	*a*PS/*i*PP ^b^ (mol/mol)	*a*PS/*i*PP ^c^ (mol/mol)	*i*PP ^c^ (mol%)	*M*_n_^d^ (g/mol)	*M*_peak_^d^ (g/mol)	*Đ*	*AN_aPS_* ^e^	*AN_iPP_* ^e^	*AN_total_* ^e^	*T*_m_^f^ (°C)	Δ*H*_m_^f^ (J/g)	*T*_c_^f^ (°C)	Δ*H*_c_^f^ (J/g)	*X*_c, *i*PP_^f^ (%)
*i*PP	-	-	-	-	6500	13,100	2.0	-	-	-	131.3	64.0	102.3	69.1	38.8
S-1	*a*PS-1	1/3	1/3.6	92.9	16,800	36,700	2.3	0.8	2.4	3.2	131.0	44.1	101.0	49.3	30.5
S-2	*a*PS-2	1/3	1/3.2	86.1	17,000	49,000	2.4	0.9	2.9	3.8	131.5	39.7	100.3	47.0	31.5
S-3	*a*PS-3	1/3	1/2.8	79.4	16,100	42,400	2.5	0.8	2.2	3.0	131.1	36.7	100.5	44.1	31.8
S-4	*a*PS-2	1/1	1/1.1	68.1	30,300	51,100	2.0	1.9	2.1	4.0	128.6	29.8	94.4	30.6	34.4

^a^ Condition of synthesis of miktoarm star copolymers: SnOct_2_ = 5 wt%, *T* = 120 °C, *t* = 3 h; ^b^ *a*PS-arm/*i*PP-arm feeding ratio; ^c^ *i*PP/*a*PS is the *a*PS-arm/*i*PP-arm ratio incorporated into the miktoarm star copolymers determined by ^1^H-NMR, and *i*PP is the propylene content in the miktoarm star copolymers determined by ^1^H-NMR; ^d^ the average molecular weight *M*_n_, and the peak molecular weight *M*_peak_ were determined by high-temperature GPC with a light-scattering detector; ^e^ in the miktoarm star copolymers, the *a*PS arm number *AN_aPS_* and the *i*PP arm number *AN_iPP_* were calculated by combining the ^1^H-NMR and GPC data, and the total arm number was *AN_total_* = *AN_aPS_* + *AN_iPP_*; ^f^ determined by DSC (Figure S2); *T*_m_, the melting point; *T*_c_, the crystalline point; ∆*H*_m_, the melting enthalpy; ∆*H*_c_, the crystalline enthalpy; *X*_c, *i*PP_, crystallinity of *i*PP calculated by calorimetry.

**Table 3:** Schematics of miktoarm star copolymers P_x_S_y_ with different lengths of soluble *a*PS arms in selective solvent DMF.

Miktoarm Stars Synthesized in This Work	
***i*PP:** 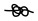	***a*PS:** 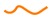
S-1 (P_2_S_1_)	S-2 (P_3_S_1_)
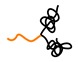	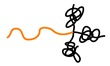
S-3 (P_2_S_1_)	S-4 (P_2_S_2_)
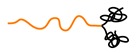	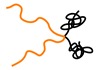

## Supplementary Materials

The following are available online at https://www.mdpi.com/2073-4360/11/10/1574/s1.
