# Supplementary material for: Isotactic-Polypropylene/Atactic-Polystyrene Miktoarm Star Copolymers: Synthesis and Aggregation Morphology
Source: Polymers (Basel). 2019 Sep 27;11(10):1574. doi: 10.3390/polym11101574 (PMC6835624; doi:10.3390/polym11101574)
Supplement: Supplementary file 1 [file polymers-11-01574-s001.pdf]

polymers

MDPI

Supporting Information

Isotactic-Polypropylene/Atactic-Polystyrene Miktoarm Star Copolymers: Synthesis and Aggregation Morphology

Yuanjie Wang, Xinzhi Liu, Liying Liu, Hui Niu*

State Key Laboratory of Fine Chemicals, Department of Polymer Science and Engineering, School of Chemical Engineering, Dalian University of Technology, Dalian 116024, China

E-mail address: hniu@dlut.edu.cn

Table S1. Purification of *i*PP/*a*PS miktoarm star copolymers [a].

Samples	Miktoarm Star Copolymer (g)	Miktoarm Star Copolymer Yield (wt %)	THF Soluble at r.t. (*a*PS wt %)	THF Insoluble under Reflux (*i*PP wt %)
**S-1**	0.460	46.0	9.0	45.0
**S-2**	0.570	57.0	11.0	32.0
**S-3**	0.550	55.0	8.5	36.5

[a] The *a*PS homopolymer was removed by THF dialyzing at room temperature (r.t.) for 24h; the *i*PP homopolymer was removed by Soxhlet extraction with boiled THF for 10 h; after the extraction the soluble part was collected to get the purified miktoarm star copolymer.

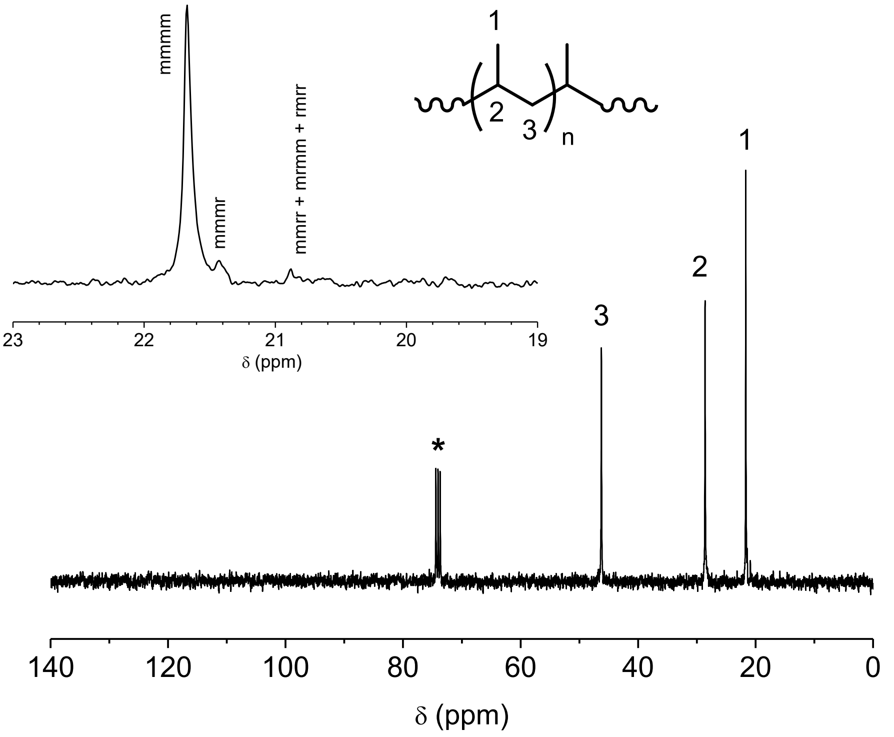

Figure S1. ^{13}C-NMR spectrum of the CH_3OSi-terminated *i*PP arm precursor.

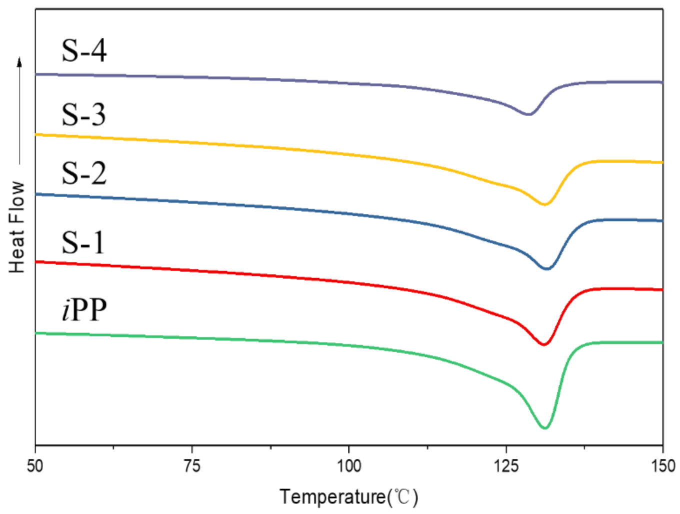

Figure S2. DSC heating curves of the *i*PP arm precursor and *i*PP/*a*PS miktoarm star copolymers (all graphics have the same ordinate units).

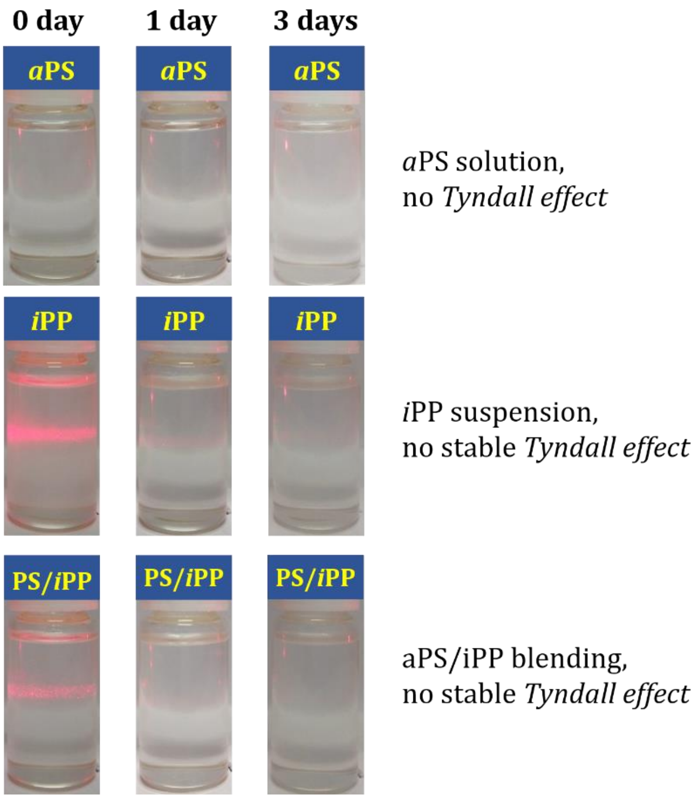

Figure S3. Photographs of *i*PP and *a*PS homopolymer and their blends solution irradiated by a laser beam.

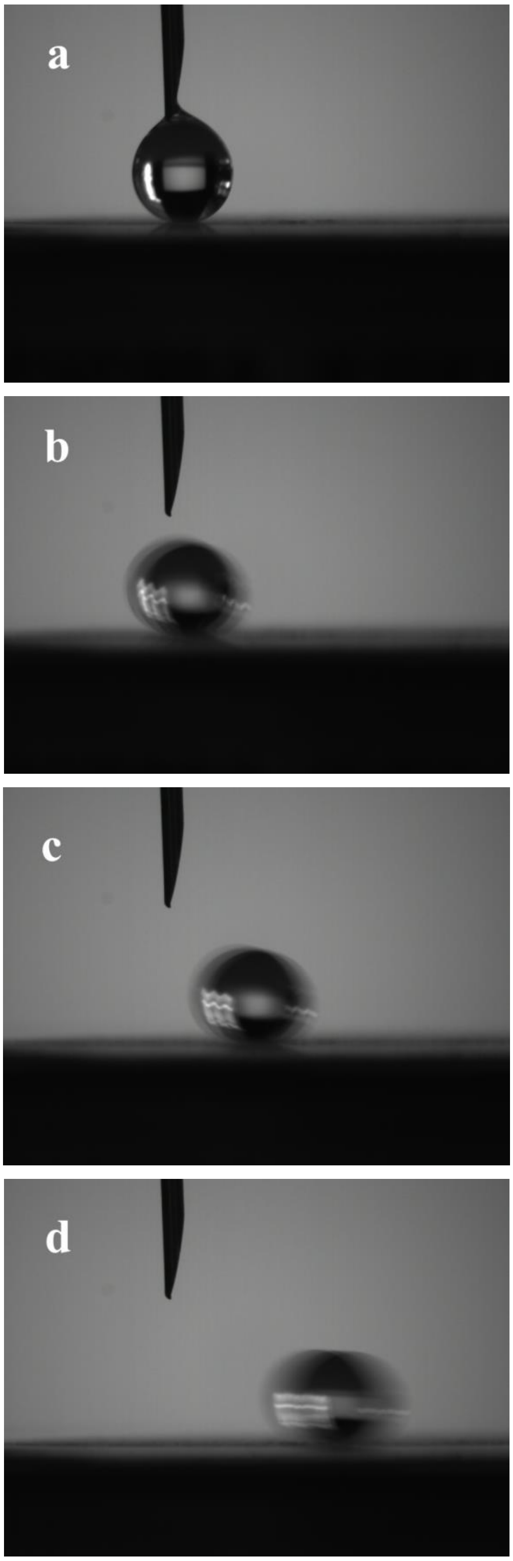

Figure S4. The rolling of water drop on the S-(2+3) surface with inclined angle of 1.5°.
